# Triglyceride-glucose index and heart failure: a systematic review and meta-analysis

**DOI:** 10.1186/s12933-023-01973-7

**Published:** 2023-09-07

**Authors:** Amirmohammad Khalaji, Amir Hossein Behnoush, Shaghayegh Khanmohammadi, Kimiya Ghanbari Mardasi, Sourena Sharifkashani, Amirhossein Sahebkar, Caterina Vinciguerra, Alessandro Cannavo

**Affiliations:** 1 School of Medicine, Tehran University of Medical Sciences, Poursina St., Keshavarz Blvd., 1417613151 Tehran, Iran; 2 Non-Communicable Diseases Research Center, Endocrinology and Metabolism Population Sciences Institute, Tehran University of Medical Sciences, Tehran, Iran; 3 Tehran Heart Center, Tehran University of Medical Sciences, Tehran, Iran; 4 Student Research Committee, Alborz University of Medical Sciences, Karaj, Iran; 5 Biotechnology Research Center, Pharmaceutical Technology Institute, Mashhad University of Medical Sciences, Mashhad, Iran; 6 Applied Biomedical Research Center, Mashhad University of Medical Sciences, Mashhad, Iran; 7 School of Medicine, The University of Western Australia, Perth, Australia; 8 Department of Biotechnology, School of Pharmacy, Mashhad University of Medical Sciences, Mashhad, Iran; 9 Department of Translational Medicine Sciences, Federico II University of Naples, Naples, Italy

**Keywords:** Heart failure, Triglycerides, Blood glucose, Insulin resistance, Systematic review, Meta-analysis

## Abstract

**Background:**

Insulin resistance (IR) is a major metabolic disorder observed in heart failure (HF) and is tightly associated with patients’ poor prognosis. The triglyceride-glucose index (TyG) has been proposed as a surrogate marker of IR in HF. Yet, whether TyG is a reliable clinical marker is still under debate. Hence, we aimed to respond to this relevant question via a systematic review and meta-analysis of existing studies.

**Methods:**

A systematic search was conducted in PubMed, Embase, Scopus, and Web of Science to find studies investigating the TyG index in patients with HF or its association with the incidence of HF. Adjusted hazard ratios (HR) and 95% confidence intervals (CI) were pooled through random-effect meta-analysis. HRs were calculated using TyG as a continuous variable (1 unit increase) and by comparing the group with the highest TyG to the lowest TyG group.

**Results:**

Thirty studies, involving 772,809 participants, were included in this systematic review. Meta-analysis of seven studies comparing the highest-TyG to the lowest-TyG group showed a significantly increased risk of HF in the former group (HR 1.21, 95% CI 1.14 to 1.29, *P* < 0.01). The same result was found when pooling the HRs for a one-unit increase in the TyG index (HR 1.17, 95% CI 1.08 to 1.26). Similarly, a more elevated TyG index was associated with a higher incidence of HF in patients with type 2 diabetes or coronary artery disease. Additionally, the incidence of adverse events (readmission and mortality) in patients with HF was associated with TyG.

**Conclusion:**

Our findings support the TyG index as a valuable marker to assess the risk of HF incidence in different populations and as a prognostic marker in patients with HF. Further studies should be conducted to confirm these associations and investigate the clinical utility of the TyG index.

**Supplementary Information:**

The online version contains supplementary material available at 10.1186/s12933-023-01973-7.

## Introduction

Despite improvements in managing heart failure (HF), this disorder still significantly burdens the global healthcare system [1, 2]. The reason why HF patients continue to worsen even if they receive optimal therapies and medical care is still unclear, demanding urgent and novel tools to help clinicians in risk stratification, diagnosis, and prognosis [3]. In this regard, several pre-clinical and clinical studies provided evidence that perturbations in cardiac and systemic metabolism can contribute to the progression of cardiac disease and a loss of pharmacological efficacy [4]. Accordingly, for over a century, a link between insulin sensitivity and resistance (IR), a typical hallmark of type 2 diabetes mellitus (T2DM), and HF has been noted [5]. Several studies have suggested a bi-directional link between these two conditions [6]. For instance, people with diabetes are more susceptible to HF than those without this syndrome. Moreover, the link between diabetes and HF resulted in stronger when ischemic heart disease was excluded [5]. Conversely, IR is associated with HF in patients without diabetes [7] and patients with HF have a high incidence of IR and are at augmented risk of developing diabetes [6]. Based on this premise, quantifying insulin sensitivity and IR in patients with/without HF is very important to predict cardiac adverse event risk and to monitor outcomes of therapeutic interventions [8]. In this sense, several methods have been developed and applied in the clinical setting, like the gold standard hyperinsulinemic-euglycemic clamp test or the homeostasis model assessment for IR (HOMA-IR). However, these tests can be challenging as they are expensive, time-consuming, burdensome, and invasive, impeding their application in the cities of undeveloped countries [9]. Therefore, more simple, dimensionless, low-cost tools to assess IR have been identified and tested, such as the triglyceride-glucose index (TyG). This index is a logarithmized product of fasting triglyceride (TG) and fasting plasma glucose (FPG), measured in routine biochemical tests [10, 11].

The TyG index is positively associated with diabetes and increased risk of metabolic and atherosclerotic cardiovascular diseases [11, 12]. Further, a positive correlation has been reported between the TyG index and the prognosis in patients with HF [13]. A higher TyG index was directly related to impaired left ventricular (LV) structure and function [14, 15], with augmented myocardial fibrosis [16], and to an increased risk of HF [14]. Notably, other studies demonstrated that the TyG index is associated with the short-term mortality rate of non-diabetic patients admitted to the hospital for acute HF (AHF) [17], or with major adverse cardiovascular events (MACEs) in patients with ischemic HF undergoing percutaneous coronary intervention (PCI) [18]. Despite these premises, the potential application of the TyG index in managing HF still needs to be entirely determined and consolidated. To this aim, we objectively merged and systematically reviewed the overall literature on the TyG index and HF.

## Methods

This systematic review and meta-analysis was registered in PROSPERO (Registration Number: CRD42023437470) and was conducted in accordance with the Preferred Reporting Item for Systematic Reviews and Meta-Analysis (PRISMA) 2020 recommendations [19].

### Search strategy

PubMed, Embase, Scopus, and Web of Science databases were systematically searched for studies published in English up to June 16, 2023. The search terms that were used included: (“triglyceride-glucose index” OR “TyG” OR “Triglyceride/glucose index” OR “triglyceride glucose”) AND (“heart failure” OR “left ventricular dysfunction” OR “heart decompensation” OR “cardiac failure” OR “myocardial failure” OR “heart decompensation”). More details about the applied search methods in each database are described in Additional file 1: Table S1.

### Study selection and eligibility criteria

In this study, we included studies that reported the TyG Index in patients older than 18 years of age who had confirmed diagnoses or were followed for incidence of HF. TyG index is calculated from TG and FPG as follows:

$$TyG=\text{ln}\left(TG\left(\frac{mg}{dL}\right) \times \frac{FPG\left(\frac{mg}{dL}\right)}{2}\right)$$

Also, we included studies that reported the association between the TyG Index and the prognosis or outcomes of HF patients. In addition, studies comparing the TyG Index with controls or different stages of HF disease were included. Finally, articles not reporting the TyG Index, animal studies, and conference abstracts were excluded from our research.

Two authors (AK and AHB) independently conducted the study assessment based on predefined eligibility criteria. They entered the initial search results in the EndNote 21 software (Thomson Reuters, New York, USA). After eliminating the duplicates, they started title and abstract screening and the full-text screening of potential articles according to the predefined criteria.

### Data extraction and quality assessment

Data extraction and quality assessment of the selected studies were accomplished by two independent authors (AK and AHB) who resolved disagreements through consensus. The following data were extracted: the first author’s name, year of publication and country of origin, sample size, mean age, sex, LV ejection fraction (LVEF), the exposure, outcomes, and adjustments. The exposure represented the TyG index level. Additionally, the outcomes included the incidence of HF, all-cause mortality, cardiovascular death, hospitalization rate, and cardiovascular complications in HF patients.

The Newcastle-Ottawa Scale (NOS) was the tool employed to evaluate the included studies’ qualities [20]. This tool is designed and suggested by the Cochrane Handbook for the assessment of observational studies’ qualities [21]. For cohort studies, the three main domains to be rated include selection, comparability, and outcome for which a maximum of four, two, and three stars can be rated. On this scale, a score of ≥ 7 is considered high quality. Two independent authors (AK and AHB) assessed the qualities and in case of disagreement, a third author (SK) resolved the issue.

### Statistical analysis

Statistical analyses were carried out using R [version 4.3.0]. We used the adjusted hazard ratios (HRs) with their corresponding 95% confidence intervals (CIs) as a general indicator to assess the association between the TyG and the incidence of HF. If the TyG index was considered a categorical variable, the HRs were calculated by comparing groups with the highest TyG index to those with the lowest TyG index. Moreover, if the TyG index was considered as a continuous variable, the HRs reflecting the risk per one-unit increment of the TyG index were calculated.

Random-effect meta-analysis (restricted maximum likelihood [REML]) was used to pool the HRs for HF incidence obtained by individual studies for comparison of the highest TyG group and the lowest one in addition to HRs for HF incidence for one unit increase in TyG index. Cochrane’s Q test and the I^2^ statistic were used to evaluate inter-study heterogeneity. I^2^ > 50% or *P* < 0.1 reflected the presence of significant heterogeneity [22]. We used the random-effects model to integrate the potential heterogeneity among the enrolled articles [23]. *P* < 0.05 was considered statistically significant throughout the analyses.

## Results

### Literature search and included studies

**Fig. 1:**
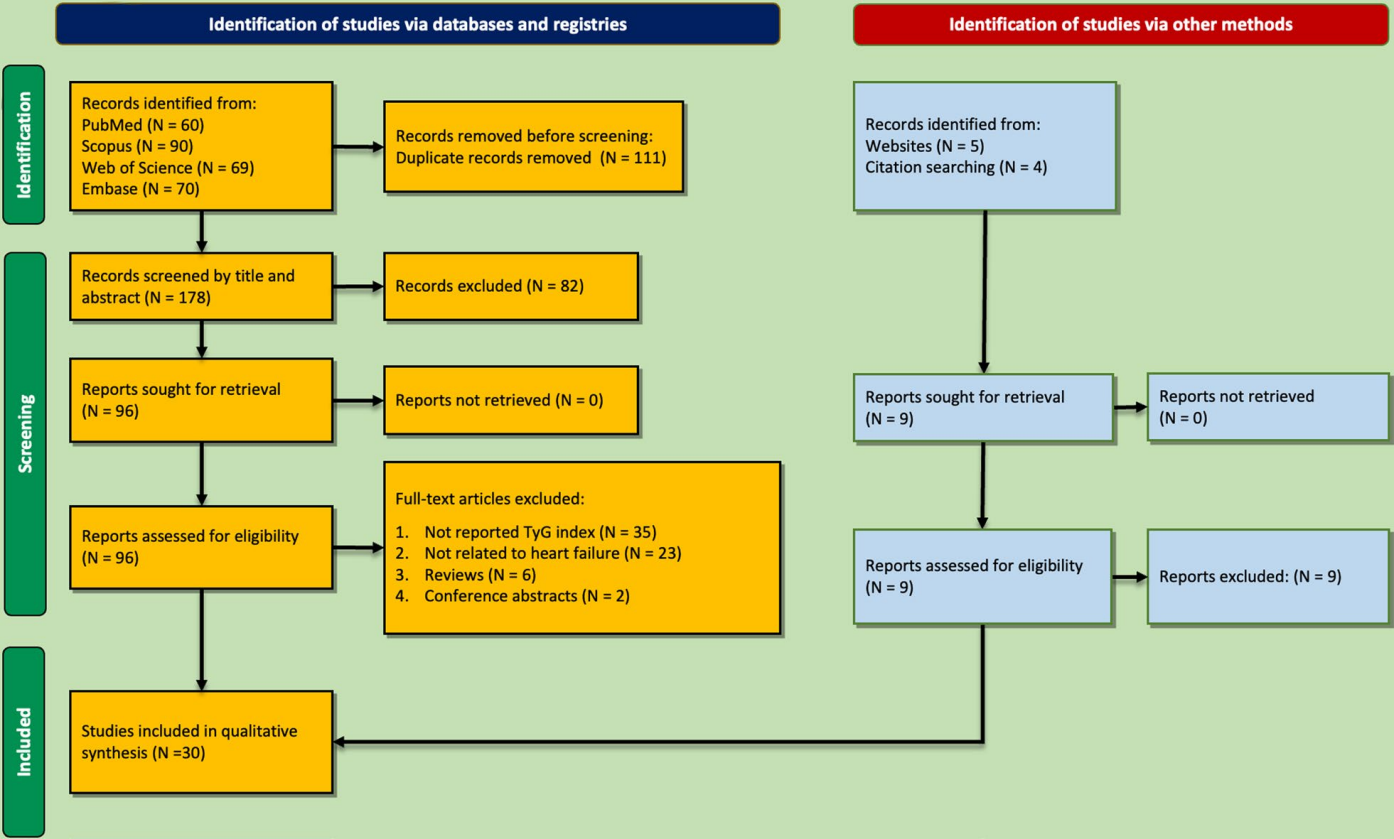
PRISMA flowchart representing the study selection process and reasons for exclusion

Two hundred eighty-nine records resulted from the initial search in four databases. Of these, 111 were excluded as they were duplicates. Afterward, 82 records were removed in the title/abstract screening step, and 66 were removed in the full-text assessment for reasons mentioned in Fig. 1. Finally, 30 studies were included in this review [9, 13–18, 24–46] and Table 1 summarizes for each study all the characteristics including population, sample size, mean age, male percentage, LVEF, TyG index, and main findings.

**Table 1:** Baseline characteristics and main findings of the included studies

Study	Year	Location	Population	Sample size	Mean age	Male (%)	LVEF (%)	TyG index	Main findings
Abuduaini et al. [24]	2023	China	Patients with cardiomyopathy and T2DM	1514	65.6 (11.1)	73.9	41.9 (7.6)	7.6 (0.8)	Increased TyG levels were associated with higher HF incidence in patients with cardiomyopathy and T2DM (HR 7.3, 95% CI 3.4 to 15.7, *P* < 0.001).
Al-Ali et al. [25]	2022	Iraq	Non-diabetic patients presenting with acute STEMI	61	54.6 (11.6)	78.7	58.5 (9.8)	4.8 (0.2)	No significant difference was found in HF incidence between tertiles of TyG (*P* = 0.09). However, LVEF was negatively correlated with the TyG index (r = -0.32, *P* = 0.01).
Chen et al. [26]	2023	China	Hospitalized patients with T2DM and LVEF ≥ 50%	150	53.4 (13.8)	64.0	60.1 (4.7)	NR	Higher quartiles of TyG were associated with left ventricular systolic dysfunction (GLS < 18%).
Cheng et al. [17]	2023	China	Non-diabetic patients with acute HF and without ACS	886	68.9 (14.8)	55.5	43.3 (10.4)	9.5 (1.3)	Acute HF patients with higher-than-median TyG levels had significantly higher mortality compared to the lower-than-median TyG group (12.6% vs. 5.6%, *P* < 0.001).
Chiu et al. [27]	2021	Taiwan	Individuals undergoing echocardiography	823	61.3 (13.1)	58.7	63.6 (13.1)	8.9 (0.3)	The higher TyG index quartile was correlated with lower LVEF (*P* = 0.003), elevated LVM (*P* = 0.081), and increased LAD (*P* = 0.004).
Gao et al. [28]	2021	China	Non-obstructive acute MI without previous revascularization	1179	55.7 (11.7)	73.5	60.5 (7.5)	8.8 (0.3)	No difference was found in terms of hospitalization for HF in tertiles of TyG (*P* = 0.081).
Guo et al. [13]	2021	China	Patients with chronic HF and T2DM	546	65.2 (12.0)	66.3	40.2 (14.9)	NR	Cardiovascular death and rehospitalization for HF were significantly higher in higher tertiles of TyG compared to tertile 1 (*P* < 0.05).
Han et al. [29]	2022	China	Patients with HF	4411	70.6 (12.6)	48.4	49.1 (10.4)	8.6 (0.7)	Higher TyG levels were associated with higher in-hospital mortality (OR 1.3, 95% CI 1.0 to 1.6, *P* = 0.024).
Huang et al. [30]	2022	China	CAD patients who underwent PCI	922	64.1 (11.0)	79.6	43.8 (13.7)	NR	Higher TyG levels were associated with higher worsening HF (HR 1.03, 95% CI 1.02 to 1.08, *P* < 0.001).
Huang et al. [14]	2022	USA	Population-based cohort aged 45 to 64 years	12,374	54.1 (5.7)	44.7	65.2 (6.7)	8.7 (0.2)	Higher TyG levels were associated with a higher risk of incident HF (HR 1.15, 95% CI 1.10 to 1.21, *P* < 0.001).
Huang et al. [31]	2022	China	Patients with acute decompensated HF	932	69.0 (14.1)	62.1	NR	NR	Comparable incidences of all-cause mortality, cardiovascular death, and MACCE were found between tertiles of TyG (*P* > 0.05).
Jung et al. [32]	2022	Korea	Cancer survivors	155,167	59.9 (12.9)	40.9	NR	8.5 (0.6)	A comparable association between HF incidence and TyG index was found (*P* > 0.05).
Li et al. [33]	2022	China & Hong Kong	Population-based adult cohort	115,341	51.4 (12.5)	79.6	NR	8.6 (0.7)	The incidence of HF was significantly higher in higher levels of TyG (*P* < 0.001).
Li et al. [9]	2023	USA	Population-based adult cohort	12,388	47.5 (0.3)	48.2	NR	8.6 (0)	Higher incidence of HF was found in patients with higher TyG levels (OR 1.34, 95% CI 1.02 to 1.76, *P* = 0.04).
Liao et al. [34]	2022	China	Hypertensive HF patients (LVEF ≥ 50%)	559	52.8 (9.6)	54.6	64.5 (8.1)	7.9 (1.0)	TyG index was significantly higher in HFpEF patients compared to non-HFpEF ones (*P* < 0.001). A significant negative correlation between LVEF and TyG index was found (r = -0.468, *P* = 0.001).
Mao et al. [35]	2019	China	Patients with NSTE-ACS	438	61.1 (11.2)	67.4	NR	8.8 (0.5)	Incidence of congestive HF was comparable between high-TyG and low-TyG groups (*P* > 0.05).
Muhammad et al. [36]	2023	Sweden	Population-based cohort	32,960	45.6 (7.4)	67.5	NR	NR	Higher TyG levels were associated with higher incidence of HF (HR 1.30, 95% CI 1.08 to 1.56, *P* < 0.01).
Sanlialp et al. [37]	2021	Turkey	Hospitalized patients with ACS	170	66.1 (13.0)	61.8	NR	9.1 (0.7)	In-hospital incidence of HF was comparable between high-TyG and low-TyG groups (*P* = 0.715).
Sanlialp et al. [38]	2021	Turkey	HF and non-HF patients without ACS	69	63.4 (9.4)	46.4	43.2 (4.1)	8.8 (0.6)	TyG index was significantly higher in patients with HF compared to non-HF controls (*P* < 0.001).
Shi et al. [39]	2022	China	Patients with HF	901	NR	44.5	50.8 (13.2)	7.8 (0.7)	No significant differences between 1- month mortality and readmission rates were found between quartiles of TyG.
Si et al. [40]	2021	UK	Population-based cohort aged 40 to 69 years	273,368	55.8 (8.0)	42.7	NR	8.6 (0.5)	Comparable rate of HF incidence between quartiles of TyG was found (*P* > 0.05).
Sun et al. [41]	2023	China	Adult patients with T2DM	183	49.2 (12.3)	70.5	61.1 (1.2)	9.3 (0.8)	TyG level were significantly higher in SLVD patients compared to non-SLVD ones (*P* < 0.001). However, LVEF were comparable between tertiles of TyG (*P* = 0.56).
Sun et al. [18]	2023	China	Patients with ischemic HF undergoing elective PCI	2055	60.3 (11.0)	82.2	40.6 (6.2)	9.0 (0.7)	Higher MACE incidence was found in higher quartiles of TyG (*P* < 0.001).
Tai et al. [42]	2022	North America	Patients with T2DM	10,196	62.8 (6.6)	61.5	NR	9.5 (0.7)	Higher MACE incidence was found in quartile 2, 3, and 4 of TyG compared to quartile 1 (*P* < 0.01). Moreover, increase in TyG was associated with higher MACE (*P* < 0.001).
Wang et al. [43]	2022	China	Patients admitted with ACS and underwent CCTA	935	65.0 (13.7)	69.0	54.4 (8.6)	NR	Patients with higher quartiles of TyG showed higher HF rehospitalization (*P* = 0.004).
Wang et al. [44]	2023	China	T2DM patients without cardiac symptoms	180	53.8 (9.2)	56.7	64.2 (2.9)	9.5 (NR)	LVEF was comparable between high-TyG and low-TyG groups (*P* = 0.09). TyG index showed an AUC of 0.706 [95% CI 0.612 to 0.801) in detecting T2DM patients with risk of HFpEF.
Xu et al. [15]	2022	China	Population-based cohort	138,620	48.5 (13.3)	79.5	NR	8.6 (0.2)	Patients in quartiles 3 and 4 of TyG showed significantly higher HF incidence compared to quartile 1 (*P* < 0.05).
Yang et al. [16]	2021	China	Hospitalized HF patients with CMR examination	103	58.3 (8.9)	68.9	48.8 (14.9)	10.0 (0.8)	The composite outcome of all-cause mortality and HF hospitalization was significantly higher in higher tertiles of TyG (*P* < 0.001).
Zeng et al. [45]	2022	USA	Population-based adult cohort	4992	25.0 (4.4)	45.5	NR	7.8 (0.5)	Higher quartiles of TyG were associated with higher congestive HF incidence (*P* < 0.001).
Zhang et al. [46]	2022	China	Patients who underwent isolated CABG with T2DM	386	66.0 (8.9)	71.5	52.4 (6.7)	9.2 (0.7)	Rehospitalization for HF was significantly higher in patients with high TyG compared to the low-TyG group (33.1% vs. 10.9%, *P* = 0.001).

*T2DM* type 2 diabetes mellitus, *TyG* triglyceride-glucose index, *HR* hazard ratio, *OR* odds ratio, *CI* confidence interval, *HF* heart failure, *LVEF* left ventricular ejection fraction, *GLS* global longitudinal strain, *NR* not reported, *ACS* acute coronary syndrome, *LVM* left ventricular mass, *LAD* left atrial diameter, *CAD* coronary artery disease, *PCI* percutaneous coronary intervention, *MACE* major adverse cardiovascular events, *MACCE* major adverse cardiac and cerebrovascular events, *NSTE-ACS* non-ST-elevation acute coronary syndrome, *CCTA* coronary computed tomography angiography, *CMR* cardiac magnetic resonance imaging

As illustrated in Additional file 1: Tables S2, the included studies had NOS scores of 6 and 7. The studies were either assessing HF incidence in population-based cohorts, T2DM patients, or coronary artery disease (CAD) patients. Also, the incidence of adverse events in patients with HF was assessed in some. For studies that reported HRs or ORs, the definitions of outcomes are available in Additional file 1: Table S3. Moreover, Additional file 1: Table S4 mentions all covariates that were used for adjustment in multivariable models.

### Association between the TyG index and incidence of outcomes

Twenty-one studies evaluated the association between the TyG index and the incidence of outcomes in their population [9, 13–18, 24, 26, 29–33, 35, 36, 39–42, 45]. Table 2 describes the difference in outcomes between high- or low-TyG index groups, tertiles (T) and quartiles (Q) of the TyG index, and the TyG index as a continuous variable. Overall, in most cases and all populations, higher TyG groups were associated with higher HR of HF incidence in adjusted models. Also, studies assessing patients with HF showed that a higher TyG index was associated with a poorer prognosis.

**Table 2:** Outcomes in different groups/levels of the TyG index

Study	Year	Population	Outcome	Group 1	Group 2	Group 3	Group 4	Group 5	Group 6	Continuous
Population-based cohorts										
Huang et al. [14]	2022	Population-based cohort aged 45 to 64 years	HF	TyG < 8.2 [Ref]	8.2 ≤ TyG < 8.6 [aHR 0.99] [95% CI 0.86 to 1.15]	8.6 ≤ TyG < 9.0 [aHR 1.08] [95% CI 0.93 to 1.25]	TyG ≥ 9.0 [aHR 1.25] [95% CI 1.08 to 1.45]^**^	–	–	Per 1-SD (0.60) increase [aHR 1.15] [95% CI 1.10 to 1.21]^***^
Jung et al. [32]	2022	Adult cancer survivor patients	HF	TyG < 8.0 [Ref]	8.0 ≤ TyG < 8.5 [aHR 0.95] [95% CI 0.81 to 1.11]	8.5 ≤ TyG < 9.0 [aHR 0.94] [95% CI 0.80 to 1.10]	9.0 ≤ TyG < 9.5 [aHR 1.06] [95% CI 0.89 to 1.26]	9.5 ≤ TyG < 10 [aHR 0.95] [95% CI 0.75 to 1.22]	TyG ≥ 10 [aHR 1.01] [95% CI 0.67 to 1.52]	–
Li et al. [33]	2022	Population-based adult cohort (Kailuan cohort)	HF	TyG ≤ 8.18 [Ref]	8.18 < TyG ≤ 8.57 [aHR 1.00] [95% CI 0.88 to 1.12]	8.57 < TyG ≤ 9.05 [aHR 1.12] [95% CI 1.00 to 1.26]^*^	TyG > 9.05 [aHR 1.23] [95% CI 1.09 to 1.39]^***^	–	–	Per 1-unit increase [aHR 1.17] [95% CI 1.10 to 1.24]^***^
Li et al. [33]	2022	Population-based adult cohort (Hong Kong cohort)	HF	TyG ≤ 6.89 [Ref]	6.89 < TyG ≤ 7.31 [aHR 1.07] [95% CI 0.92 to 1.23]	7.31 < TyG ≤ 7.80 [aHR 1.17] [95% CI 1.01 to 1.35]^*^	TyG > 7.80 [aHR 1.21] [95% CI 1.04 to 1.40]^**^	–	–	Per 1-unit increase [aHR 1.13] [95% CI 1.05 to 1.22]^**^
Li et al. [9]	2023	Population-based adult cohort	HF	TyG < 8.12 [Ref]	8.12 ≤ TyG < 8.55 [aOR 0.91] [95% CI 0.52 to 1.56]	8.55 ≤ TyG < 9.00 [aOR 1.13] [95% CI 0.71 to 1.80]	TyG ≥ 9.00 [aOR 1.45] [95% CI 0.87 to 2.41]	–	–	Per 1-unit increase [aOR 1.34] [95% CI 1.02 to 1.76]^*^
Muhammad et al. [36]	2023	Population-based cohort	HF	TyG < 4.38	4.38 ≤ TyG < 4.55 [aHR 1.04] [95% CI 0.91 to 1.18]	4.55 ≤ TyG < 4.74 [aHR 1.00] [95% CI 0.88 to 1.15]	TyG ≥ 4.74 [aHR 1.12] [95% CI 0.97 to 1.29]	–	–	Per 1-unit increase [aHR 1.30] [95% CI 1.08 to 1.56]^**^
Si et al. [40]	2020	Population-based cohort aged 40 to 69 years	HF	Q1 [Ref]	Q2 [OR 1.05] [95% CI 0.93 to 1.18]	Q3 [OR 1.12] [95% CI 1.00 to 1.27]	Q4 [OR 1.11] [95% CI 0.98 to 1.25]	–	–	–
Xu et al. [15]	2022	Population-based cohort	HF	TyG < 8.16 [Ref]	8.16 ≤ TyG < 8.55 [aHR 0.95] [95% CI 0.81 to 1.10]	8.55 ≤ TyG < 9.00 [aHR 1.01] [95% CI 0.87 to 1.18]	TyG ≥ 9.00 [aHR 1.24] [95% CI 1.07 to 1.44]^*^	–	–	–
Zeng et al. [45]	2022	Population-based adult cohort	HF	Q1 (median 7.3) [Ref]	Q2 (median 7.7) [aHR 1.2] [95% CI 0.5 to 3.1]	Q3 (median 8.0) [aHR 1.7] [95% CI 0.7 to 4.1]	Q4 (median 8.4) [aHR 3.4] [95% CI 1.4 to 8.0]^***^	–	–	–
Type 2 diabetes										
Abuduaini et al. [24]	2023	Cardiomyopathy and T2DM	HF	TyG ≤ 7.21 [Ref]	7.21 < TyG < 7.89 [aHR 2.66] [95% CI 1.16 to 6.07]*	TyG ≥ 7.89 [aHR 7.33] [95% CI 3.42 to 15.7]^***^	–	–	–	–
Chen et al. [26]	2023	Hospitalized patients with T2DM and LVEF ≥ 50%	GLS < 18%	TyG ≤ 8.89 [Ref]	8.89 < TyG < 9.44 [aHR 1.28] [95% CI 0.30 to 5.55]	9.44 < TyG < 9.83 [aHR 4.52] [95% CI 1.12 to 18.3]^*^	TyG > 9.83 [aHR 5.23] [95% CI 1.12 to 24.5]^*^	–	–	–
Guo et al. [13]	2021	Patients with chronic HF and T2DM	CV death or HF rehospitalization	TyG < 8.55 [Ref]	8.55 ≤ TyG < 9.06 [aHR 1.66] [95% CI 1.02 to 2.70]^*^	TyG ≥ 9.06 [aHR 2.46] [95% CI 1.51 to 4.01]^*^	–	–	–	–
Sun et al. [41]	2023	Adult patients with T2DM	SLVD	–	–	–	–	–	–	Per 1-unit increase [aOR 1.61] [95% CI 1.00 to 2.59]^*^
Tai et al. [42]	2022	Patients with T2DM	Fatal or hospital HF	TyG ≤ 9.00 [Ref]	9.00 < TyG ≤ 9.47 [aHR 1.15] [95% CI 0.91 to 1.45]	9.47 < TyG ≤ 9.95 [aHR 1.05] [95% CI 0.91 to 1.20]	TyG > 9.95 [aHR 1.17] [95% CI 1.07 to 1.29]^**^	–	–	Per 1-SD increase [aHR 1.25] [95% CI 1.11 to 1.40]^***^
Coronary artery disease										
Huang et al. [30]	2022	CAD patients who underwent PCI	Worsening HF	TyG ≤ 8.51 [Ref]	8.51 < TyG ≤ 8.98 [aHR 1.31] [95% CI 0.82 to 2.07]	TyG > 8.98 [aHR 2.44] [95% CI 1.59 to 3.72]^***^		–	–	Per 0.1-unit increase [aHR 1.07] [95% CI 1.05 to 1.10]^***^
Mao et al. [35]	2019	Patients with NSTE-ACS	CHF	–	–	–	–	–	–	Per 1-unit increase [aHR 0.41] [95% CI 0.08 to 2.06]
Sun et al. [18]	2023	Patients with ischemic HF undergoing elective PCI	MACE	TyG < 8.54 [Ref]	8.54 ≤ TyG < 8.93 [aHR 1.31] [95% CI 1.02 to 1.68]^*^	8.93 ≤ TyG < 9.41 [aHR 1.71] [95% CI 1.34 to 2.18]^***^	TyG ≥ 9.41 [aHR 1.92] [95% CI 1.48 to 2.49]^***^	–	–	Per 1-unit increase [aHR 1.41] [95% CI 1.22 to 1.62]^***^
Heart failure										
Cheng et al. [17]	2023	Non-diabetic patients with acute HF and without ACS	In-hospital mortality	TyG ≤ 9.44 [Ref]	TyG > 9.44 [aOR 1.89] [95% CI 1.13 to 3.47]^*^	–	–	–	–	^–^
Han et al. [29]	2022	Patients with HF	In-hospital mortality	TyG < 8.25 [Ref]	8.25 ≤ TyG < 8.78 [aOR 1.54] [95% CI 1.00 to 2.35]^*^	TyG ≥ 8.78 [aOR 2.08] [95% CI 1.28 to 3.35]^**^	–	–	–	Per 1-unit increase [aOR 1.89] [95% CI 1.42 to 2.50]^***^
Huang et al. [31]	2022	Patients with acute decompensated HF	All-cause mortality	TyG < 8.83 [Ref]	8.83 ≤ TyG < 9.32 [aHR 1.07] [95% CI 0.67 to 1.74]	TyG ≥ 9.32 [aHR 2.09] [95% CI 1.23 to 3.55]^**^	–	–	–	–
Shi et al. [39]	2022	Patients with HF	HF readmission	TyG < 7.36 [aOR 1.65] [95% CI 1.09 to 2.45]^*^	Q2 [Ref]	Q3 [aOR 1.18] [95% CI 0.71 to 1.80]	Q4 [aOR 1.12] [95% CI 0.72 to 1.82]	–	–	–
Yang et al. [16]	2021	Hospitalized HF patients with CMR examination	Mortality or HF hospitalization	–	–	–	–	–	–	Per 1-SD increase [aHR 2.01] [95% CI 1.01 to 4.01]^*^

*T2DM* type 2 diabetes mellitus, *HF* heart failure, Ref: reference, *TyG index* triglyceride-glucose index,* HR* hazard ratio, *OR* odds ratio, *CI* confidence interval, *LVEF* left ventricular ejection fraction, *GLS* global longitudinal strain, *ACS* acute coronary syndrome, *CV* cardiovascular, *CAD* coronary artery disease, *PCI* percutaneous coronary intervention, *CH*F congestive heart failure, *SLVD* subclinical left ventricular dysfunction, *MACE* major adverse cardiovascular events,* CMR* cardiac magnetic resonance imaging

^*^ *P* < 0.05, ^**^P < 0.01, ^***^P < 0.001

#### Population-based cohorts

Using data from the Atherosclerosis Risk in Communities (ARIC) study, Huang et al. [14] evaluated the association between the TyG index and the risk of incident HF in a large population-based cohort (12,374 participants). Comparing the difference among Q of the baseline TyG index, these authors observed that participants in the highest Q (Q4; mean: 9.5 + 0.4) had a greater risk of incident HF compared to those in the Q1 (mean: 8.0 + 0.2) (*P* < 0.001). Moreover, a 1 SD (0.60) increase in the TyG index was associated with a higher incidence of HF in this population (*P* < 0.001).

Significantly, two independent reports also confirmed these results. Xu and coworkers [15] demonstrated that in a population-based cohort of 138,620 participants, Q4 of TyG (9.00-11.65) was significantly associated with a higher HF incidence compared with Q1 (6.77–8.16) (*P* < 0.05). Analogously, Zeng et al. [45] reported that in a total of 4992 participants enrolled in the Coronary Artery Risk Development in Young Adults (CARDIA) investigation [from 1985 to 1986 (year 0)], only those participants in the Q4 of TyG (8.3–8.7) were at an increased risk of HF events than those in the Q1 (7.1–7.4) throughout the clinical monitoring timeframe (log-rank test, *P* < 0.001).

In another study, Li and coworkers [33], through analyzing two large Chinese cohorts (total of 115,341 subjects), demonstrated that a high TyG index was an independent and causal risk factor for incident HF. These authors found higher HF incidence with every one-unit increase in TyG index in both cohorts assessed (*P* < 0.001). Moreover, they observed that Q2 (8.19–8.57), Q3 (8.58–9.05), and Q4 (9.06–12.51) of the TyG index were associated with higher HF incidence compared with Q1 (3.60–8.18) in both cohorts (*P* < 0.001). For their part, Li and coworkers [9] analyzed the dataset from The Nation Health and Nutrition Examination Survey (NHANES) (2009–2018). This study analyzed 12,388 subjects, including 322 (2.6%) individuals with HF, and interestingly while no differences in HF incidence were found among Q of TyG, a one-unit increase in the TyG index was associated with significantly higher HF incidence (*P* = 0.04). Indeed, subjects in Q4 of the TyG index (≥ 9) had a significantly higher prevalence of HF (OR 1.41; 95% CI 1.01–1.95) compared to those in lower Q (1–3; < 9).

According to this observation, Muhammad et al. [36] found a significant increase in HF incidence with every one-unit increase in the TyG index (*P* < 0.001). However, no difference among Q of TyG in terms of HF incidence using a fully adjusted model was observed (*P* > 0.05). These results were similarly reported by Si and colleagues [40], who failed to find a significant difference in HF incidence among Q of the TyG index (*P* > 0.05), and by Jung et al. [32], comparing the incidence of HF in cancer survivors classified according to the TyG index, found no significant difference between these groups in most comparisons (*P* > 0.05).

**Fig. 2:**
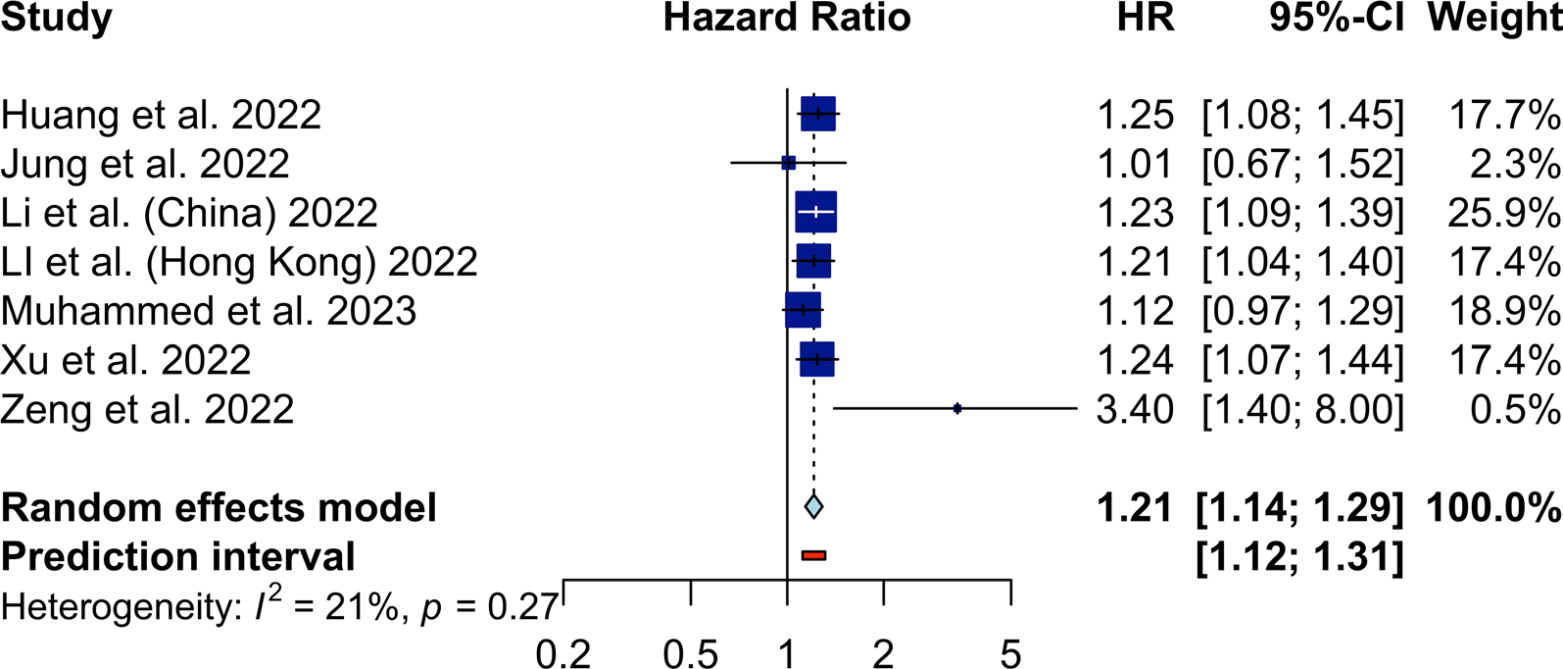
Forest plot showing the random-effect meta-analysis of HF incidence in comparison of the highest TyG group vs. lowest TyG group

**Fig. 3:**
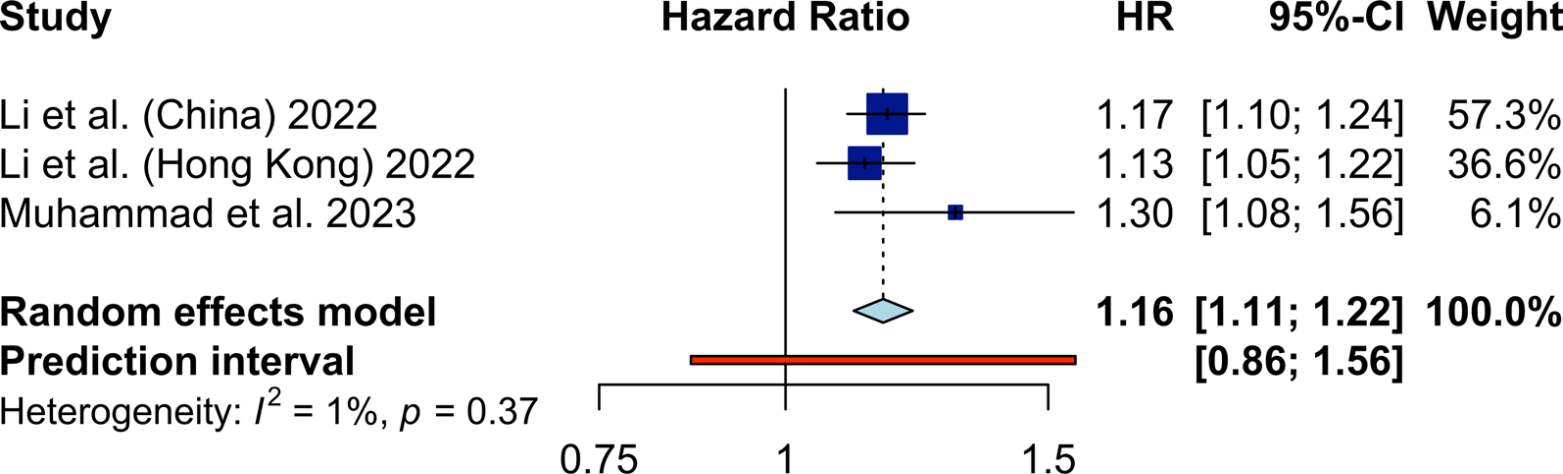
Forest plot showing the random-effect meta-analysis of HF incidence using the TyG index as a continuous variable (1-unit increase)

Next, we performed a meta-analysis to compare the highest TyG group with the lowest (reference) group in each study, and as shown in Fig. 2, pooling the adjusted HRs of individual studies indicated a significantly higher incidence of HF (HR 1.21, 95% CI 1.14 to 1.29, *P* < 0.01; I^2^ 21%). Another meta-analysis was also performed to assess the association of every 1-unit increase in the TyG index in normal populations on the incidence of HF. The forest plot, shown in Fig. 3, demonstrated that a one-unit increase in TyG was significantly associated with the HR of HF (HR 1.17, 95% CI 1.08 to 1.26).

#### TyG and T2DM

We next sought to determine the relationship between the TyG index, diabetes, and HF. In this regard, Abuduaini et al. [24] analyzed 1514 individuals divided into three groups based on their TyG index T (T = ≤ 7.21; T2 = 7.21–7.89; T3 = ≥ 7.89) with ischemic cardiomyopathy and T2DM and reported that participants in T2 and T3 presented a significant increase in the incidence of HF, compared to those in the T1. In line with these data, Guo et al. [13] compared the composite outcome of cardiovascular death or HF rehospitalization in patients (a total of 546) with chronic HF and T2DM and found a higher incidence in T2 (TyG index ≥ 8.55 and < 9.06) and T3 (TyG index ≥ 9.06) of the TyG index compared to the reference (T1; TyG index < 8.55).

For their part, Chen et al. [26] used a global longitudinal strain (GLS) < 18% to detect subclinical LV systolic dysfunction (SLVD) in patients with T2DM and found a significantly higher rate of GLS < 18% in Q3 and Q4 of the TyG index compared to the reference range (TyG ≤ 8.89). Next, Sun et al. [41] found that in a small cohort of 183 patients with T2DM, a one-unit increase in the TyG index was associated with higher SLVD incidence (OR 1.61, *P* < 0.05).

Finally, Tai et al. [42], in 10,196 patients with T2DM, found a positive association between a one-SD increase in the TyG index and HF incidence in patients with T2DM (*P* < 0.001). Interestingly, the authors observed that MACE incidence was increased in participants with a higher Q of TyG index than in Q1 (*P* < 0.05). Moreover, the Q4 of TyG was associated with higher HF incidence than the Q1.

#### Coronary artery disease (CAD) and TyG

To assess the association between IR and short-term outcomes of acute ST-elevation myocardial infarction (STEMI) in patients without T2DM, Al-Ali et al. [25] conducted a cross-sectional study of 61 patients. These authors divided the patients into three groups based on TyG index T (T1 = < 4.73, T2 = 4.73–4.87, and T3 ≥ 4.87) and found that patients in the T3 presented significantly higher 4-week mortality (30%), compared to those in the lower T (T1). However, despite a significant negative correlation between the TyG index and LVEF (R = − 0.32, *P* = 0.01), there was a higher likelihood among the T of having LVEF < 55% and a non-successful revascularization rate. In analogy with this observation, Gao and colleagues [28] compared hospitalization for HF in patients with non-obstructive MI between T of the TyG index and found comparable incidence (*P* = 0.081). Conversely, Huang et al. [30], including patients with significant mitral regurgitation, observed that those in T3 of TyG index showed higher worsening HF compared to the reference (T1; *P* < 0.001). For their part, Mao et al. [35] in an observational study that included 438 consecutive patients with non-ST elevation acute coronary syndrome (NSTE-ACS) evaluated the association between the TyG index and the incidence of HF. Interestingly, despite these, authors found that patients with higher TyG values had a higher incidence of diabetes (*P* < 0.001), glucose metabolism disorder (*P* < 0.001), metabolic syndrome (*P* < 0.001), and higher MACE (*P* = 0.006), while no increase in CHF incidence (*P* = 0.280) was detected with one unit increase in the TyG index. Similar results were also provided by Sanlialp et al. [37], in hospitalized patients with ACS, failed to find significant differences between high-TyG (> 9.04) and low-TyG (≤ 9.04) in terms of HF incidence (37% vs. 35%, *P* = 0.715).

Conversely, Huang et al. [30], in their analysis of patients with mitral regurgitation, observed that those in T3 of TyG index showed higher worsening HF compared to the reference (*P* < 0.001). Finally, Sun et al. [18] assessed the incidence of MACE in patients with ischemic HF undergoing PCI and found that those in Q2, Q3, and Q4 had significantly increased aHR of MACE compared with the first Q.

#### The TyG index and outcomes in HF

To examine whether and how TyG can be a useful prognostic indicator for HF patients, Cheng and coworkers [17] examined 886 out of 1620 non-diabetic patients with AHF. Interestingly, after adjusting for age, comorbidities, and HF etiologies, these authors reported significantly higher in-hospital mortality in those patients with higher values of TyG index [OR 1.89, 95% CI 1.13 to 3.47, *P* = 0.023]. On the same line, in a retrospective study, Han et al. [29] evaluated in-hospital mortality in 4411 patients diagnosed with HF and divided them into three groups based on the TyG index T (T1 to T3). Significantly, they found higher mortality in patients in T2 and T3 of the TyG index compared to those in T1. Notably, a one-unit increase in the TyG index was significantly associated with higher in-hospital mortality (*P* < 0.001). Similar results were also observed by Huang and colleagues [31]. Indeed, in 932 patients with acute decompensated HF (ADHF), these authors reported that, regardless of their diabetic status, the TyG index was independently associated with poor prognosis as subjects in T3 (≥ 9.32) of the TyG index had more all-cause mortality than the reference. In line with this data, Yang et al. [16] found a significant positive association between an increase in TyG index and incidence of all-cause mortality or HF hospitalization (*P* = 0.047) in patients with HF. Further, Guo et al. [13] found that in patients with CHF and T2DM, the incidence of rehospitalization due to HF in the T3 group was significantly higher than that observed in the T1 group (HR 1.84, 95% CI 1.16 to 2.91). Finally, Shi and colleagues [39], in 901 HF patients, examined the usage of the TyG index as a predictor of the incidence of readmission of HF patients. To this aim, they divided patients into four Q (Q1 to Q4) based on the TyG index and, surprisingly, found that a lower TyG index (< 7.36) independently augmented the risk of 6-month readmission (*P* = 0.024).

### Diagnostic and prognostic ability of TyG index

Seven studies assessed the diagnostic and prognostic performance of the TyG index in diagnosing HF or predicting outcomes in patients with HF [16, 17, 26, 34, 38, 44, 45]. In this regard, Chen et al. [26] divided 150 T2DM patients with preserved LVEF (≥ 50%) into four TyG indexes Q, and found that the higher TyG index had acceptable utility in predicting GLS < 18% (area under the receiver operating characteristic curve [AUC]: 0.678, *P* < 0.001). Next, Cheng et al. [17] found an AUC of 0.688 [95% CI 0.631 to 0.745] for the TyG index in predicting in-hospital mortality in nondiabetic patients with AHF. For their part, Liao et al. [34] used the TyG index to predict HF with preserved EF (HFpEF) in patients with essential hypertension. To this aim, these authors enrolled 559 hypertensive patients (273 with HFpEF and 286 without HFpEF) and found an OR of 2.924 [95% CI 1.945 to 4.395] for the TyG index in the prediction of HFpEF. The AUC for discriminating HFpEF from non-HFpEF patients using the TyG index was 0.778 [95% CI 0.707 to 0.849]. In another study, Sanlialp et al. [38] evaluated the diagnostic role of the TyG index in discriminating patients with HF from non-HF individuals. Of note, they demonstrated that the TyG index was significantly higher in HF patients (9.11 ± 0.59 vs. 8.55 ± 0.55, *P* < 0.001) and reported an AUC of 0.745 (71% sensitivity, 51% specificity, *P* < 0.001) for HF diagnosis. Next, Wang et al. [44] aimed at identifying patients with suspicious or positive HFpEF using the TyG index. Their model had an AUC of 0.706 [95% CI 0.612 to 0.801] in patients with T2DM but without cardiac symptoms. In patients with HF, Yang et al. [16] found an AUC of 0.709 [95% CI 0.611 to 0.794] for the TyG index in predicting events (all-cause mortality and HF hospitalization). Finally, Zeng et al. [45], in a population-based cohort of adults (aged between 18 and 30 years), found an AUC of 0.675 [95% CI 0.604 to 0.746] for the TyG index in predicting the incident risk of CHF.

### LVEF in different groups of TyG index in patients with HF

Five studies included patients with HF and compared LVEF between groups of patients classified based on the TyG index as a cut-off [13, 17, 31, 39, 41]. Cheng et al. [17] found comparable LVEF values between low-TyG (TyG ≤ 9.44) and high-TyG (TyG > 9.44) groups in nondiabetic patients with AHF (*P* = 0.169). In another report, Guo et al. [13] found lower LVEF in the upper TyG T subgroup of patients with chronic HF and T2DM, compared to the lower TyG T (*P* = 0.014). Next, Huang et al. [31], in their study, included patients with ADHF and found a significantly higher prevalence of LVEF ≤ 40% in the T3 of the TyG index (≥ 9.32) compared with lower T (T1 [< 8.83] and T2 [8.83–9.32]). Shi et al. [39] found significantly different LVEF levels among quartiles of TyG in patients with HF, while the observed trend was U-shaped (higher in the Q1 and Q4 of the TyG index). Finally, Sun et al. [41] compared LVEF among Q of the TyG index in patients with ischemic HF who underwent elective PCI and found no significant difference among these groups (*P* = 0.187).

## Discussion

In this report, we performed a comprehensive systematic review and meta-analysis on a total of 772,809 individuals assessed in 30 studies (Table 1) to investigate the association between the TyG index and the incidence of HF and outcomes in patients with HF. Interestingly, the principal findings of this study are that: (1) regardless of the presence of T2DM and HF, a higher TyG index equals more adverse outcomes (increased mortality, hospitalization rates, cardiovascular events, and reduced LVEF); (2) the TyG index has demonstrated diagnostic ability in distinguishing HF patients from non-HF individuals; (3) the TyG index could serve as a simple and cost-effective marker for risk stratification and early detection of individuals at higher risk for HF.

HF represents the end stage of most cardiovascular diseases. Despite the enormous progress in therapy and tools development in the last decades to predict the incidence and adverse outcomes, HF prevalence continues to rise dramatically over time [47]. Therefore, identifying more specific predictors of future HF events represents one of the most significant achievements of the research in the field. In this sense, several biomarkers have been identified, tested, and implemented in clinical practice along with prediction models for HF that primarily rely on traditional risk factors [48], including T2DM and IR [49, 50].

Several methods to assess IR have been developed, and among these, the HOMA-IR and the TyG index are considered valuable and reliable markers of IR [9]. HOMA-IR, as one of the most commonly used indices, is calculated by using fasting glucose and insulin [51]. However, this index presents several limitations that make it unavailable in most laboratories in developing countries [9]. The TyG index is less expensive and easily available, representing an ideal and valid alternative for identifying IR in normal populations [52, 53]. Moreover, HOMA-IR showed limitations in evaluating IR in low-BMI T2DM patients that had β-cell malfunction and insulin secretory defects [54]. In a Brazilian population, Vasques et al. reported that the TyG index outperformed the HOMA-IR [53]. Also, in the Korean population, the TyG index was superior to HOMA-IR in the prediction of metabolic syndrome with cutoffs of 8.718 and 1.8, respectively [55]. Finally, in a study conducted on Chinese diabetic patients with high BMI, TyG (cutoff 7.99) was able to identify IR more effectively, compared to HOMA-IR (cutoff 3.39) [56].

In addition, as demonstrated in this study, including the TyG index in the prediction models of HF could sensibly improve their accuracy in determining individuals at risk for HF among the general population. Importantly, the strict association discussed throughout this study between the TyG index and HF outcomes underscores the potential role of IR in the pathogenesis of HF [57]. Indeed, IR is a well-recognized factor with multiple noxious effects, including endothelial dysfunction, oxidative stress, and myocardial remodeling, all contributing to impaired cardiac function and the development and progression of HF [33, 58, 59].

Of course, several discrepancies have been observed among the studies included here. Some of these may be attributed to differences in study populations, designs, adjustments for confounding factors, sample sizes, and applied cut-off values which could negatively influence the association of TyG or increase the risk of readmission of HF. The use of different cutoffs among the studies was inevitable since most of the studies categorized the patients based on tertiles and quartiles of the TyG index which are different among the countries, settings, and populations. Hence, there was no universal cutoff available for this index and we compared the groups within each study with each other. This necessitates the need to identify the populations and the characteristics of patients in each region and provide a local model for each setting. Since this index is easily calculated, it seems feasible for researchers to provide these cutoffs. Moreover, the need to adjust and provide a valid and reliable method to use in different settings seems unavoidable.

Additionally, our meta-analysis revealed that an increase of one unit in the TyG index raised the risk of HF development in normal populations (HR 1.16, 95% CI 1.11 to 1.22). Furthermore, the meta-analysis comparing the group with the highest TyG index to the lowest TyG index group showed a higher incidence of HF (HR 1.21, 95% CI 1.14 to 1.29).

The clinical implications of TyG in the management of HF patients could be one of our main practical findings in this study. Since the TyG index could predict HF incidence in different populations from diabetics to those with CAD, it can be added to the routine clinical assessments of individuals at risk of HF. Moreover, its clinical use in predicting adverse events in patients with HF will add value to this index. HF clinics in countries and settings with limited resources can benefit from this easily measured index and stratify the risk of patients, which eventually leads to better care and recommendations given to the patients.

## Strengths and limitations

Our study was the first to determine the relationship between the TyG index and HF. Moreover, the high number of studies included can provide good evidence and clues for further research. In this study, we studied this association in a variety of populations from CAD to diabetics. Finally, providing the comparison of the diagnostic ability of TyG can provide useful data for researchers. On the other hand, this study has five main limitations that need to be mentioned. First, the cut-off values for the TyG index were different across the included studies, which can lead to differences in classifying individuals into high or low TyG index groups, potentially affecting the observed associations with HF outcomes. Second, different study populations and sample sizes may also affect the results. Third, differences in adjustment for confounding factors and failure to adequately adjust for these factors can lead to biased estimates of the association. Fourth, most existing studies are observational, which limits their ability to establish causality. Lastly, comparing and combining the results from different studies was challenging due to using different outcome measures. These limitations contribute to the mixed findings and highlight the need for further research to clarify the relationship between the TyG index and HF outcomes.

## Conclusion

Overall, this study supports the TyG index as an easy-to-use diagnostic and prognostic surrogate marker of IR and HF events to be implemented in clinical practice. As our analysis shows, this index is associated with HF incidence and outcomes in all groups of patients, and monitoring the TyG index might be beneficial in patients with established HF as it predicts adverse events. Further studies and correlation with some of the parameters primarily influencing IR lifestyle modifications (diet and exercise), pharmacological interventions, and other confounding factors (e.g., comorbidities) are warranted to validate these findings.

### Supplementary Information

**Additional file 1: Table S1.** The search queries used for each database and the search results. **Table S2.** Qualities of included studies based on NOS. **Table S3.** Definition of outcomes. **Table S4.** Adjusted covariates in multivariable models.

## Data Availability

Not applicable.
