# Supplementary material for: Triglyceride-glucose index and heart failure: a systematic review and meta-analysis
Source: Cardiovasc Diabetol. 2023 Sep 7;22:244. doi: 10.1186/s12933-023-01973-7 (PMC10486123; doi:10.1186/s12933-023-01973-7)
Supplement: Supplementary file 1 — Additional file 1: Table S1. The search queries used for each database and the search results. Table S2. Qualities of included studies based on NOS. Table S3. Definition of outcomes. Table S4. Adjusted covariates in multivariable models. [file 12933_2023_1973_MOESM1_ESM.docx]

**Supplementary Materials**

***Supplementary Table 1.*** *The search queries used for each database and the search results*

| Query | | Results (No.)  16 June 2023 |
| --- | --- | --- |
| **PubMed** | | |
| #1 | (“lipid ind*”[tiab] OR “TyG”[tiab] OR “triglyceride glucose”[tiab] OR “triglyceride-glucose”[tiab] OR “triglyceride-glucose index”[tiab]) | 3,265 |
| #2 | (“heart failure”[tiab] OR “cardiac failure”[tiab] OR “myocardial failure”[tiab] OR “heart decompensation”[tiab] OR “decompensation, heart”[tiab] OR “left ventricular dysfunction”[tiab] OR "Heart Failure"[Mesh]) | 270,087 |
| #3 | #1 AND #2 | 60 |
| **Embase** | | |
| #1 | (“lipid ind*”:ti,ab,kw OR “TyG”:ti,ab,kw OR “triglyceride glucose”:ti,ab,kw OR “triglyceride-glucose”:ti,ab,kw OR “triglyceride-glucose index”:ti,ab,kw) | 4,375 |
| #2 | (“heart failure”:ti,ab,kw OR “cardiac failure”:ti,ab,kw OR “myocardial failure”:ti,ab,kw OR “heart decompensation”:ti,ab,kw OR “left ventricular dysfunction”:ti,ab,kw) | 388,376 |
| #3 | #1 AND #2 | 70 |
| **Web of Science** | | |
| #1 | (TS=“lipid ind*” OR TS=“TyG” OR TS=“triglyceride glucose” OR TS=“triglyceride-glucose” OR TS=“triglyceride-glucose index”) | 3,697 |
| #2 | (TS=“heart failure” OR TS=“cardiac failure” OR TS=“myocardial failure” OR TS=“heart decompensation” OR TS=“left ventricular dysfunction”) | 311,983 |
| #3 | #1 AND #2 | 69 |
| **SCOPUS** | | |
| #1 | (TITLE-ABS-KEY(“lipid ind*”) OR TITLE-ABS-KEY(“TyG”) OR TITLE-ABS-KEY(“triglyceride glucose”) OR TITLE-ABS-KEY(“triglyceride-glucose”) OR TITLE-ABS-KEY(“triglyceride-glucose index”)) | 5,804 |
| #2 | (TITLE-ABS-KEY(“heart failure”) OR TITLE-ABS-KEY(“cardiac failure”) OR TITLE-ABS-KEY(“myocardial failure”) OR TITLE-ABS-KEY(“heart decompensation”) OR TITLE-ABS-KEY(“left ventricular dysfunction”)) | 407,374 |
| #3 | #1 AND #2 | 90 |
| ***TOTAL RECORDS*** | | ***289*** |
| ***TOTAL RECORDS AFTER REMOVING DUPLICATES*** | | ***178*** |

***Supplementary Table 2.*** *Qualities of included studies based on NOS*

| **Study** | **Selection** | | | | **Comparability** | **Outcome** | | | **Overall**  **Score** |
| --- | --- | --- | --- | --- | --- | --- | --- | --- | --- |
|  | **Representation of exposed cohort** | **Selection of the non-exposed cohort** | **Ascertainment of exposure** | **Outcome of interest presence** |  | **Assessment of outcome** | **Sufficient length of follow-up** | **Loss to follow-up** |  |
| **Abuduaini et al. (2023)** | * | * | * | * | - | * | *  (55.4 months) | * | 7 |
| **Al-Ali et al. (2022)** | * | * | * | * | - | * | *  (4 weeks) | * | 7 |
| **Chen et al. (2023)** | * | * | * | * | - | * | - | * | 6 |
| **Cheng et al. (2023)** | * | * | * | * | - | * | *  (In-hospital) | * | 7 |
| **Chiu et al. (2021)** | * | * | * | * | - | * | - | * | 6 |
| **Gao et al. (2021)** | * | * | * | * | - | * | *  (41.7 months) | * | 7 |
| **Guo et al. (2021)** | * | * | * | * | - | * | *  (12 months) | * | 7 |
| **Han et al. (2022)** | * | * | * | * | - | * | *  (In-hospital) | * | 7 |
| **Huang et al. (2022)** | * | * | * | * | - | * | *  (3.7 years) | * | 7 |
| **Huang et al. (2022)** | * | * | * | * | - | * | *  (22.5 years) | * | 7 |
| **Huang et al. (2022)** | * | * | * | * | - | * | *  (478 days) | * | 7 |
| **Jung et al. (2022)** | * | * | * | * | - | * | *  (10 years) | * | 7 |
| **Li et al. (2022)** | * | * | * | * | - | * | *  (12.3 years) | * | 7 |
| **Li et al. (2023)** | * | * | * | * | - | * | - | * | 6 |
| **Liao et al. (2022)** | * | * | * | * | * | * | - | * | 7 |
| **Mao et al. (2019)** | * | * | * | * | - | * | *  (12 months) | * | 7 |
| **Muhammad et al. (2023)** | * | * | * | * | - | * | *  (16.9 years) | * | 7 |
| **Sanlialp et al. (2021)** | * | * | * | * | - | * | *  (In-hospital) | * | 7 |
| **Sanlialp et al. (2021)** | * | * | * | * | * | * | - | * | 7 |
| **Shi et al. (2022)** | * | * | * | * | - | * | *  (6 months) | * | 7 |
| **Si et al. (2020)** | * | * | * | * | - | * | - | * | 6 |
| **Sun et al. (2023)** | * | * | * | * | - | * | - | * | 6 |
| **Sun et al. (2023)** | * | * | * | * | - | * | *  (36 months) | * | 7 |
| **Tai et al. (2022)** | * | * | * | * | - | * | *  (8.8 years) | * | 7 |
| **Wang et al. (2022)** | * | * | * | * | - | * | *  (34.5 months) | * | 7 |
| **Wang et al. (2023)** | * | * | * | * | - | * | - | * | 6 |
| **Xu et al. (2022)** | * | * | * | * | - | * | *  (8.8 years) | * | 7 |
| **Yang et al. (2021)** | * | * | * | * | - | * | *  (12.3 months) | * | 7 |
| **Zeng et al. (2022)** | * | * | * | * | - | * | *  (31 years) | * | 7 |
| **Zhang et al. (2022)** | * | * | * | * | - | * | *  (60 months) | * | 7 |

***Supplementary Table 3.*** *Definition of outcomes*

| **Study** | **Year** | **Population** | **Outcome** | **Exact definition of the outcome** |
| --- | --- | --- | --- | --- |
| **Population-based cohorts** | | | | |
| Huang et al. (1) | 2022 | Population-based cohort aged 45 to 64 years | HF | First HF hospitalization or death from HF |
| Jung et al. (2) | 2022 | Adult cancer survivor patients | HF | Hospitalization due to HF |
| Li et al. (3) | 2022 | Population-based adult cohort (Kailuan cohort) | HF | HF was primarily diagnosed according to European Society of Cardiology guidelines |
| Li et al. (3) | 2022 | Population-based adult cohort (Hong Kong cohort) | HF | HF events were identified using ICD-9 codes |
| Li et al. (4) | 2023 | Population-based adult cohort | HF | Patients were considered as having HF if they answered “yes” to this question: “*Has a doctor or other health professional ever told you that you had congestive HF?*” |
| Muhammad et al. (5) | 2023 | Population-based cohort | HF | Incident HF |
| Si et al. (6) | 2020 | Population-based cohort aged 40 to 69 years | HF | HF events were identified using ICD-9 and ICD-10 codes |
| Xu et al. (7) | 2022 | Population-based cohort | HF | Newly diagnosed HF events diagnosed on the basis of clinical symptoms, echocardiography, chest radiography, and electrocardiography according to European Society of Cardiology guidelines |
| Zeng et al. (8) | 2022 | Population-based adult cohort | HF | Congestive HF diagnosed by a physician |
| **Type 2 diabetes** | | | | |
| Abuduaini et al. (9) | 2023 | Cardiomyopathy and T2DM | HF | Newly diagnosed HF as a component of MACCE |
| Chen et al. (10) | 2023 | Hospitalized patients with T2DM and LVEF ≥50% | GLS <18% | SLVD using the predefined cutoff of GLS < 18% according to European Association of Cardiovascular Imaging |
| Guo et al. (11) | 2021 | Patients with chronic HF and T2DM | Cardiovascular death or HF rehospitalization | Composite outcome of cardiovascular death or rehospitalization due to HF |
| Sun et al. (12) | 2023 | Adult patients with T2DM | SLVD | SLVD using the predefined cutoff of GLS < 18% according to European Association of Cardiovascular Imaging |
| Tai et al. (13) | 2022 | Patients with T2DM | Fatal or hospital HF | Fatal or hospitalized CHF (fatal CHF was defined as death due to clinical, radiological or postmortem evidence of CHF without clinical or postmortem evidence of an acute ischemic event; hospitalized CHF was defined as participant with documented clinical and radiological evidence) |
| **Coronary artery disease** | | | | |
| Huang et al. (14) | 2022 | CAD patients who underwent PCI | Worsening HF | Unplanned rehospitalization or unscheduled physician office/emergency department visits due to HF and unplanned mitral valve surgery |
| Mao et al. (15) | 2019 | Patients with NSTE-ACS | CHF | CHF in a 12-month follow-up |
| Sun et al. (16) | 2023 | Patients with ischemic HF undergoing elective PCI | MACE | MACE including all-cause mortality, non-fatal myocardial infarction (fourth universal definition of myocardial infarction), and any revascularization. |
| **Heart failure** | | | | |
| Cheng et al. (17) | 2023 | Non-diabetic patients with acute HF and without ACS | In-hospital mortality | All-cause in-hospital mortality |
| Han et al. (18) | 2022 | Patients with HF | In-hospital mortality | All-cause in-hospital mortality |
| Huang et al. (19) | 2022 | Patients with acute decompensated HF | All-cause mortality | All-cause death including cardiovascular death (fatal stroke, myocardial infarction, congestive HF, malignant arrythmia, or other functional or structural cardiac diseases) and non-cardiovascular death |
| Shi et al. (20) | 2022 | Patients with HF | HF readmission | 6-month HF readmission |
| Yang et al. (21) | 2021 | Hospitalized HF patients with CMR examination | Mortality or HF hospitalization | Composite outcome of all-cause death or HF rehospitalization |

HF: heart failure, ICD: international classification of diseases, T2DM: type 2 diabetes mellitus, MACCE: major adverse cardiac and cerebrovascular events, LVEF: left ventricular ejection fraction, GLS: global longitudinal strain, SLVD: subclinical left ventricular dysfunction, CHF: congestive heart failure, CAD: coronary artery disease, PCI: percutaneous coronary intervention, NSTE-ACS: non-ST elevation-acute coronary syndrome, MACE: major adverse cardiovascular events, ACS: acute coronary syndrome, CMR: cardiac magnetic resonance imaging

**Supplementary Table 3 references:**

1. Huang RH, Lin YF, Ye XM, Zhong XB, Xie PH, Li MH, et al. Triglyceride-glucose index in the development of heart failure and left ventricular dysfunction: analysis of the ARIC study. EUROPEAN JOURNAL OF PREVENTIVE CARDIOLOGY. 2022;29(11):1531-41.

2. Jung MH, Yi SW, An SJ, Yi JJ, Ihm SH, Han S, et al. Associations between the triglyceride-glucose index and cardiovascular disease in over 150,000 cancer survivors: a population-based cohort study. Cardiovasc Diabetol. 2022;21(1):52.

3. Li X, Chan JSK, Guan B, Peng S, Wu X, Lu X, et al. Triglyceride-glucose index and the risk of heart failure: Evidence from two large cohorts and a mendelian randomization analysis. Cardiovasc Diabetol. 2022;21(1):229.

4. Li X, Wang J, Niu L, Tan Z, Ma J, He L, et al. Prevalence estimates of the insulin resistance and associated prevalence of heart failure among United Status adults. BMC Cardiovasc Disord. 2023;23(1):294.

5. Muhammad IF, Bao X, Nilsson PM, Zaigham S. Triglyceride-glucose (TyG) index is a predictor of arterial stiffness, incidence of diabetes, cardiovascular disease, and all-cause and cardiovascular mortality: A longitudinal two-cohort analysis. Front Cardiovasc Med. 2022;9:1035105.

6. Si S, Li J, Li Y, Li W, Chen X, Yuan T, et al. Causal Effect of the Triglyceride-Glucose Index and the Joint Exposure of Higher Glucose and Triglyceride With Extensive Cardio-Cerebrovascular Metabolic Outcomes in the UK Biobank: A Mendelian Randomization Study. Frontiers in Cardiovascular Medicine. 2021;7.

7. Xu L, Wu M, Chen S, Yang Y, Wang Y, Wu S, et al. Triglyceride-glucose index associates with incident heart failure: A cohort study. Diabetes Metab. 2022;48(6):101365.

8. Zeng X, Han D, Zhou H, Xue Y, Wang X, Zhan Q, et al. Triglyceride-Glucose Index and Homeostasis Model Assessment-Insulin Resistance in Young Adulthood and Risk of Incident Congestive Heart Failure in Midlife: The Coronary Artery Risk Development in Young Adults Study. Front Cardiovasc Med. 2022;9:944258.

9. Abuduaini B, Yang L, Jiamali N, Seyiti Z, Shan XF, Gao XM. Predictive Effect of Triglyceride-Glucose Index on Adverse Prognostic Events in Patients with Type 2 Diabetes Mellitus and Ischemic Cardiomyopathy. Diabetes Metab Syndr Obes. 2023;16:1093-107.

10. Chen YY, Fu JF, Wang Y, Zhang Y, Shi M, Wang C, et al. Association between triglyceride glucose index and subclinical left ventricular systolic dysfunction in patients with type 2 diabetes. LIPIDS IN HEALTH AND DISEASE. 2023;22(1).

11. Guo W, Zhao L, Mo F, Peng C, Li L, Xu Y, et al. The prognostic value of the triglyceride glucose index in patients with chronic heart failure and type 2 diabetes: A retrospective cohort study. Diabetes Res Clin Pract. 2021;177:108786.

12. Sun QC, Liu J, Meng R, Zhang N, Yao J, Yang F, et al. Association of the triglyceride-glucose index with subclinical left ventricular dysfunction in type 2 diabetes mellitus patients: A retrospective cross-sectional study. J Diabetes Investig. 2023.

13. Tai S, Fu L, Zhang N, Zhou Y, Xing Z, Wang Y. Impact of Baseline and Trajectory of Triglyceride-Glucose Index on Cardiovascular Outcomes in Patients With Type 2 Diabetes Mellitus. Front Endocrinol. 2022;13.

14. Huang H, Li Q, Liu J, Qiao L, Chen S, Lai W, et al. Association between triglyceride glucose index and worsening heart failure in significant secondary mitral regurgitation following percutaneous coronary intervention. Cardiovasc Diabetol. 2022;21(1):260.

15. Mao Q, Zhou D, Li Y, Wang Y, Xu SC, Zhao XH. The Triglyceride-Glucose Index Predicts Coronary Artery Disease Severity and Cardiovascular Outcomes in Patients with Non-ST-Segment Elevation Acute Coronary Syndrome. Dis Markers. 2019;2019:6891537.

16. Sun T, Huang X, Zhang B, Ma M, Chen Z, Zhao Z, et al. Prognostic significance of the triglyceride-glucose index for patients with ischemic heart failure after percutaneous coronary intervention. Front Endocrinol (Lausanne). 2023;14:1100399.

17. Cheng H, Huang W, Huang X, Miao W, Huang Y, Hu Y. The triglyceride glucose index predicts short-term mortality in non-diabetic patients with acute heart failure. Adv Clin Exp Med. 2023.

18. Han S, Wang C, Tong F, Li Y, Li Z, Sun Z, et al. Triglyceride glucose index and its combination with the Get with the Guidelines-Heart Failure score in predicting the prognosis in patients with heart failure. Front Nutr. 2022;9:950338.

19. Huang R, Wang Z, Chen J, Bao X, Xu N, Guo S, et al. Prognostic value of triglyceride glucose (TyG) index in patients with acute decompensated heart failure. Cardiovasc Diabetol. 2022;21(1):88.

20. Shi L, Liu J, Zhu X, Li T, Wen J, Wang X, et al. Triglyceride Glucose Index Was a Predictor of 6-Month Readmission Caused by Pulmonary Infection of Heart Failure Patients. Int J Endocrinol. 2022;2022:1131696.

21. Yang S, Du Y, Liu Z, Zhang R, Lin X, Ouyang Y, et al. Triglyceride-Glucose Index and Extracellular Volume Fraction in Patients With Heart Failure. Front Cardiovasc Med. 2021;8:704462.

***Supplementary Table 4.*** *Adjusted covariates in multivariable models*

| **Study** | **Year** | **Population** | **Outcome** | **Covariates** |
| --- | --- | --- | --- | --- |
| **Population-based cohorts** | | | | |
| Huang et al. (1) | 2022 | Population-based cohort aged 45 to 64 years | HF | Age, race, sex, smoking status, drinking status, hypertension, diabetes, anti-hypertensive medication, lipid-lowering medication, body mass index, estimated glomerular filtration rate, systolic blood pressure, and low-density lipoprotein cholesterol |
| Jung et al. (2) | 2022 | Adult cancer survivor patients | HF | Age, sex, household income, behavioral factors (alcohol consumption, smoking habit, and physical activity), and cardiometabolic factors (systolic blood pressure, body mass index, lipid-lowering medication use, low-density lipoprotein cholesterol, and high-density lipoprotein cholesterol) |
| Li et al. (3) | 2022 | Population-based adult cohort (Kailuan cohort) | HF | Age, gender, education, income, physical activity, smoking status, alcohol intake, diabetes, low-density lipoprotein cholesterol, high-density lipoprotein cholesterol, systolic blood pressure, diastolic blood pressure, body mass index, estimated glomerular filtration rate, high sensitivity C-reactive protein, anti-hypertensive drugs, anti-diabetes drugs, and lipid-lowering drugs |
| Li et al. (3) | 2022 | Population-based adult cohort (Hong Kong cohort) | HF | Age, sex, hypertension, diabetes mellitus, chronic kidney disease, dyslipidemia, antihypertensives, anti-diabetic drugs, antiplatelets, and lipid-lowering drugs |
| Li et al. (4) | 2023 | Population-based adult cohort | HF | Age, gender, race, marital status, body mass index, education status, low-density lipoprotein cholesterol, estimated glomerular filtration rate, uric acid, diabetes mellitus, hypertension, dyslipidemia, stroke, chronic kidney disease, family monthly poverty level category, moderate physical activity, sedentary, and current smoking |
| Muhammad et al. (5) | 2023 | Population-based cohort | HF | Age, sex, body mass index, systolic blood pressure, cholesterol, smoking status, diabetes, antihypertensive medication, physical activity, and alcohol |
| Si et al. (6) | 2020 | Population-based cohort aged 40 to 69 years | HF | Age, sex, body mass index, smoke status, fasting time, and low-density lipoprotein cholesterol |
| Xu et al. (7) | 2022 | Population-based cohort | HF | Age, sex, education, physical activity, smoking, alcohol drinking, salt intake, waist circumference, low-density lipoprotein cholesterol, high-sensitivity C-reactive protein, hypertension, and estimated glomerular filtration rate |
| Zeng et al. (8) | 2022 | Population-based adult cohort | HF | Age, sex, race, education, obesity, smoking status, hypertension, diabetes mellitus, hypercholesteremia, chronic kidney disease, low-density lipoprotein cholesterol, and high-density lipoprotein cholesterol |
| **Type 2 diabetes** | | | | |
| Abuduaini et al. (9) | 2023 | Cardiomyopathy and T2DM | HF | Age, body mass index, neutrophil, lymphocyte, platelets, uric acid, high-density lipoprotein cholesterol, low-density lipoprotein cholesterol, alkaline phosphatase, left ventricular ejection fraction, gender, total cholesterol, hypertension history, ethnicity, and medications (antiplatelet agent, angiotensin-converting enzyme inhibitors/angiotensin receptor blockers, beta-blocker, statins, oral hypoglycemic agents, and insulin) |
| Chen et al. (10) | 2023 | Hospitalized patients with T2DM and LVEF ≥50% | GLS <18% | Age, gender, diabetes duration, systolic blood pressure, hemoglobin A1c, body mass index, hypertension, heart rate, logarithmic microalbuminuria, left ventricular ejection fraction, and insulin therapy |
| Guo et al. (11) | 2021 | Patients with chronic HF and T2DM | Cardiovascular death or HF rehospitalization | Age, sex, body mass index, systolic blood pressure, diastolic blood pressure, heart rate, C-reactive protein, estimated glomerular filtration rate, N-terminal prohormone of brain natriuretic peptide, hemoglobin A1c, left ventricular ejection fraction, left ventricular end-diastolic diameter, renal insufficiency, atrial fibrillation, New York Heart Association functional classification, and the use of beta-blockers, digoxin, angiotensin-converting enzyme inhibitors/angiotensin receptor blockers/angiotensin receptor neprilysin inhibitor, spironolactone, sodium-glucose contrasporter-2 inhibitor, and metformin |
| Sun et al. (12) | 2023 | Adult patients with T2DM | SLVD | Age, sex, body mass index, systolic blood pressure, and low-density lipoprotein cholesterol |
| Tai et al. (13) | 2022 | Patients with T2DM | Fatal or hospital HF | Age, sex, previous cardiovascular event, race, body mass index, education, systolic blood pressure, diastolic blood pressure, estimated glomerular filtration rate, hemoglobin A1c, total cholesterol, low-density lipoprotein cholesterol, live alone, duration of diabetes and depression, and treatment with statins, biguanide, aspirin, angiotensin-converting enzyme inhibitors/angiotensin receptor blockers, and insulin |
| **Coronary artery disease** | | | | |
| Huang et al. (14) | 2022 | CAD patients who underwent PCI | Worsening HF | Age, gender, smoking history, body mass index, left ventricular ejection fraction, hyperlipidemia, hypertension, diabetes mellitus, anemia, chronic kidney disease, acute myocardial infarction, atrial fibrillation, renin-angiotensin-aldosterone system inhibitor, beta-blockers, loop diuretics, and mineralocorticoid receptor antagonist |
| Mao et al. (15) | 2019 | Patients with NSTE-ACS | CHF | Age, gender, metabolic syndrome, low-density lipoprotein cholesterol, high-density lipoprotein cholesterol, SYNTAX score, C-reactive protein, basal insulin, sulfonylurea, metformin, α-glucosidase inhibitor, angiotensin-converting enzyme inhibitors/angiotensin receptor blockers, beta-blocker, and percutaneous coronary intervention/coronary artery bypass grafting |
| Sun et al. (16) | 2023 | Patients with ischemic HF undergoing elective PCI | MACE | Age, sex, heart rate, body mass index, New York Heart Association class, prior percutaneous coronary intervention, platelet, albumin, total cholesterol, low-density lipoprotein cholesterol, high-density lipoprotein cholesterol, potassium, uric acid, left ventricular ejection fraction, angiotensin receptor blockers, thiazide diuretics, spironolactone, sacubitril/valsartan, diffuse lesion, SYNTAX score, left main coronary artery disease, in-stent restenosis, target vessel (left main), complete revascularization |
| **Heart failure** | | | | |
| Cheng et al. (17) | 2023 | Non-diabetic patients with acute HF and without ACS | In-hospital mortality | Age, comorbidities of cardiogenic shock, atrial fibrillation, chronic obstructive pulmonary disease, use of vasoactive drugs, and heart failure etiologies including coronary heart disease, hypertensive heart disease, any cardiomyopathy, and any valvular heart disease |
| Han et al. (18) | 2022 | Patients with HF | In-hospital mortality | Age, sex, New York Heart Association grading, heart rate, systolic blood pressure, albumin, total bilirubin, low-density lipoprotein cholesterol, blood urea nitrogen, creatinine, uric acid, hemoglobin, sodium, troponin I, N-terminal prohormone of brain natriuretic peptide, left ventricular ejection fraction, and the history of coronary artery disease, hypertension, atrial fibrillation, diabetes mellitus, chronic obstructive pulmonary disease, smoking, angiotensin-converting enzyme inhibitors/angiotensin receptor blockers/angiotensin receptor neprilysin inhibitor, beta-blockers, diuretic, and aldosterone antagonists |
| Huang et al. (19) | 2022 | Patients with acute decompensated HF | All-cause mortality | Age, gender, body mass index, systolic blood pressure, diastolic blood pressure, heart rate, hemoglobin A1c, C-reactive protein, hematocrit, red blood cell distribution width, brain natriuretic peptide, sodium, albumin, creatinine, uric acid, high-density lipoprotein cholesterol, left ventricular ejection fraction, history of hypertension, ischemic cardiomyopathy, diabetes mellitus, valvular heart diseases, atrial fibrillation, and hyperlipidemia, and use of statins, aldosterone antagonist, digoxin, diuretics, beta-blockers, antiplatelet agent, angiotensin-converting enzyme inhibitors/angiotensin receptor blockers/angiotensin receptor neprilysin inhibitor, insulin, sodium-glucose contrasporter-2 inhibitor, and metformin |
| Shi et al. (20) | 2022 | Patients with HF | HF readmission | Age, gender, body mass index, New York Heart Association, myocardial infarction, congestive heart failure, hypertension, peripheral artery disease, chronic obstructive pulmonary disease, diabetes mellitus, chronic kidney disease, left ventricular ejection fraction, left ventricular end-diastolic diameter, angiotensin-converting enzyme inhibitors/angiotensin receptor blockers, mineralocorticoid receptor antagonists, estimated glomerular filtration rate, high-sensitivity C-reactive protein, brain natriuretic peptide, albumin, cholesterol, and low-density lipoprotein cholesterol |
| Yang et al. (21) | 2021 | Hospitalized HF patients with CMR examination | Mortality or HF hospitalization | Age, sex, diabetes, hypertension, smoking, body mass index, hemoglobin A1c, triglyceride, left ventricular ejection fraction, estimated glomerular filtration rate, N-terminal prohormone of brain natriuretic peptide, high-sensitivity C-reactive protein, and extracellular volume fraction |

HF: heart failure, ICD: international classification of diseases, T2DM: type 2 diabetes mellitus, MACCE: major adverse cardiac and cerebrovascular events, LVEF: left ventricular ejection fraction, GLS: global longitudinal strain, SLVD: subclinical left ventricular dysfunction, CHF: congestive heart failure, CAD: coronary artery disease, PCI: percutaneous coronary intervention, NSTE-ACS: non-ST elevation-acute coronary syndrome, MACE: major adverse cardiovascular events, ACS: acute coronary syndrome, CMR: cardiac magnetic resonance imaging

**Supplementary Table 4 references:**

1. Huang RH, Lin YF, Ye XM, Zhong XB, Xie PH, Li MH, et al. Triglyceride-glucose index in the development of heart failure and left ventricular dysfunction: analysis of the ARIC study. EUROPEAN JOURNAL OF PREVENTIVE CARDIOLOGY. 2022;29(11):1531-41.

2. Jung MH, Yi SW, An SJ, Yi JJ, Ihm SH, Han S, et al. Associations between the triglyceride-glucose index and cardiovascular disease in over 150,000 cancer survivors: a population-based cohort study. Cardiovasc Diabetol. 2022;21(1):52.

3. Li X, Chan JSK, Guan B, Peng S, Wu X, Lu X, et al. Triglyceride-glucose index and the risk of heart failure: Evidence from two large cohorts and a mendelian randomization analysis. Cardiovasc Diabetol. 2022;21(1):229.

4. Li X, Wang J, Niu L, Tan Z, Ma J, He L, et al. Prevalence estimates of the insulin resistance and associated prevalence of heart failure among United Status adults. BMC Cardiovasc Disord. 2023;23(1):294.

5. Muhammad IF, Bao X, Nilsson PM, Zaigham S. Triglyceride-glucose (TyG) index is a predictor of arterial stiffness, incidence of diabetes, cardiovascular disease, and all-cause and cardiovascular mortality: A longitudinal two-cohort analysis. Front Cardiovasc Med. 2022;9:1035105.

6. Si S, Li J, Li Y, Li W, Chen X, Yuan T, et al. Causal Effect of the Triglyceride-Glucose Index and the Joint Exposure of Higher Glucose and Triglyceride With Extensive Cardio-Cerebrovascular Metabolic Outcomes in the UK Biobank: A Mendelian Randomization Study. Frontiers in Cardiovascular Medicine. 2021;7.

7. Xu L, Wu M, Chen S, Yang Y, Wang Y, Wu S, et al. Triglyceride-glucose index associates with incident heart failure: A cohort study. Diabetes Metab. 2022;48(6):101365.

8. Zeng X, Han D, Zhou H, Xue Y, Wang X, Zhan Q, et al. Triglyceride-Glucose Index and Homeostasis Model Assessment-Insulin Resistance in Young Adulthood and Risk of Incident Congestive Heart Failure in Midlife: The Coronary Artery Risk Development in Young Adults Study. Front Cardiovasc Med. 2022;9:944258.

9. Abuduaini B, Yang L, Jiamali N, Seyiti Z, Shan XF, Gao XM. Predictive Effect of Triglyceride-Glucose Index on Adverse Prognostic Events in Patients with Type 2 Diabetes Mellitus and Ischemic Cardiomyopathy. Diabetes Metab Syndr Obes. 2023;16:1093-107.

10. Chen YY, Fu JF, Wang Y, Zhang Y, Shi M, Wang C, et al. Association between triglyceride glucose index and subclinical left ventricular systolic dysfunction in patients with type 2 diabetes. LIPIDS IN HEALTH AND DISEASE. 2023;22(1).

11. Guo W, Zhao L, Mo F, Peng C, Li L, Xu Y, et al. The prognostic value of the triglyceride glucose index in patients with chronic heart failure and type 2 diabetes: A retrospective cohort study. Diabetes Res Clin Pract. 2021;177:108786.

12. Sun QC, Liu J, Meng R, Zhang N, Yao J, Yang F, et al. Association of the triglyceride-glucose index with subclinical left ventricular dysfunction in type 2 diabetes mellitus patients: A retrospective cross-sectional study. J Diabetes Investig. 2023.

13. Tai S, Fu L, Zhang N, Zhou Y, Xing Z, Wang Y. Impact of Baseline and Trajectory of Triglyceride-Glucose Index on Cardiovascular Outcomes in Patients With Type 2 Diabetes Mellitus. Front Endocrinol. 2022;13.

14. Huang H, Li Q, Liu J, Qiao L, Chen S, Lai W, et al. Association between triglyceride glucose index and worsening heart failure in significant secondary mitral regurgitation following percutaneous coronary intervention. Cardiovasc Diabetol. 2022;21(1):260.

15. Mao Q, Zhou D, Li Y, Wang Y, Xu SC, Zhao XH. The Triglyceride-Glucose Index Predicts Coronary Artery Disease Severity and Cardiovascular Outcomes in Patients with Non-ST-Segment Elevation Acute Coronary Syndrome. Dis Markers. 2019;2019:6891537.

16. Sun T, Huang X, Zhang B, Ma M, Chen Z, Zhao Z, et al. Prognostic significance of the triglyceride-glucose index for patients with ischemic heart failure after percutaneous coronary intervention. Front Endocrinol (Lausanne). 2023;14:1100399.

17. Cheng H, Huang W, Huang X, Miao W, Huang Y, Hu Y. The triglyceride glucose index predicts short-term mortality in non-diabetic patients with acute heart failure. Adv Clin Exp Med. 2023.

18. Han S, Wang C, Tong F, Li Y, Li Z, Sun Z, et al. Triglyceride glucose index and its combination with the Get with the Guidelines-Heart Failure score in predicting the prognosis in patients with heart failure. Front Nutr. 2022;9:950338.

19. Huang R, Wang Z, Chen J, Bao X, Xu N, Guo S, et al. Prognostic value of triglyceride glucose (TyG) index in patients with acute decompensated heart failure. Cardiovasc Diabetol. 2022;21(1):88.

20. Shi L, Liu J, Zhu X, Li T, Wen J, Wang X, et al. Triglyceride Glucose Index Was a Predictor of 6-Month Readmission Caused by Pulmonary Infection of Heart Failure Patients. Int J Endocrinol. 2022;2022:1131696.

21. Yang S, Du Y, Liu Z, Zhang R, Lin X, Ouyang Y, et al. Triglyceride-Glucose Index and Extracellular Volume Fraction in Patients With Heart Failure. Front Cardiovasc Med. 2021;8:704462.
